# Efficacy of different methanolic plant extracts on anti-methanogenesis, rumen fermentation and gas production kinetics *in vitro*

**Published:** 2012-08-15

**Authors:** S.K. Sirohi, N. Goel, P. Pandey

**Affiliations:** *Nutrition Biotechnology Lab, Animal Biotechnology Center, National Dairy Research Institute, Karnal-132001, Haryana, India*

**Keywords:** Digestible dry matter, Gas production rate constant, Methane, Methanolic plant extracts, *In vitro* gas production technique

## Abstract

The present study was carried out to evaluate the effect of methanolic extracts of three plants, mehandi (*Lawsonia inermis*), jaiphal (*Myristica fragrans*) and green chili (*Capsicum annuum*) on methanogenesis, rumen fermentation and fermentation kinetic parameters by *in vitro* gas production techniques. Single dose of each plant extract (1 ml / 30 ml buffered rumen fluid) and two sorghum fodder containing diets (high and low fiber diets) were used for evaluating the effect on methanogenesis and rumen fermentation pattern, while sequential incubations (0, 1, 2, 3, 6 9, 12, 24, 36, 48, 60, 72 and 96 h) were carried out for gas production kinetics. Results showed that methane production was reduced, ammonia nitrogen was increased significantly, while no significant effect was found on pH and protozoal population following addition of different plant extracts in both diets except mehandi. Green chili significantly reduced digestibility of dry matter, total fatty acid and acetate concentration at incubation with sorghum based high and low fiber diets. Among all treatments, green chili increased potential gas production, while jaiphal decreased the gas production rate constant significantly. The present results demonstrate that methanolic extracts of different plants are promising rumen modifying agents. They have the potential to modulate the methane production, potential gas production, gas production rate constant, dry matter digestibility and microbial biomass synthesis.

## Introduction

Methane and ammonia are energetically wasteful and harmful end products of rumen fermentation process in ruminants. These end products also create ecological problems as methane is a more potent greenhouse gas than carbon dioxide.

Hence, the recent goal of ruminant microbiologists and nutritionists is to manipulate the ruminal microbial ecosystem to improve the feed conversion efficiency and reduce methane production. Much of the research in the past decades has focused on the effects of antimicrobial compounds on ruminal fermentation mainly on ionophores and antibiotics (Russell, 1987).

These compounds seem to inhibit hydrogen-producing microorganisms and Gram-positive lactate-producing bacteria such as *Streptococcus bovis* (Russell, 1987; Russell and Strobel, 1989).

Antibiotics have been banned by European Union’s Agricultural ministry since the first of January 2006, due to the risk of the presence of its residue in milk and meat and its effects on human health. Therefore the attention of animal nutritionist community has recently shifted towards the use of natural antimicrobial or bioactive plant secondary metabolites (PSM) as a safe means of ruminal fermentation modulators. PSM possess antimicrobial activity that is highly specific, which raises their possibility to target methanogens and modulate ruminal fermentation to improve nutrient utilization in ruminants (Hristov *et al.*, 1999).

Herbal preparations have been used for centuries for various purposes because of their antimicrobial properties (Davidson and Naidu, 2000) and because most of them are categorized under GRAS (Generally Recognized as Safe) for human consumption (FDA, 2004). The use of herbal preparations appears as one of the most natural alternatives to the antibiotic use in animal nutrition.

The aim of this study was to determine the effects of mehandi (*Lawsonia inermis*), jaiphal (*Myristica fragrans*) and green chili (*Capsicum annuum*) methanolic extracts on methanogenesis and gas production kinetics using *in vitro* technique.

## Materials and Methods

### Procedure of plant extracts preparation

Three plant parts; mehandi leaves (*Lawsonia inermis*, T_1_), jaiphal fruits (*Myristica fragrans*, T_2_) and green chili (*Capsicum annuum*, T_3_) were used for the preparation of methanolic extracts. Jaiphal and green chili were purchased from a local market, while mehandi leaves were manually collected from National Dairy Research Institute, Karnal, India. Jaiphal fruits and mehandi leaves were dried overnight in hot air oven at 70°C and ground in mills to pass a 1 mm sieve. Finely-grounded jaiphal fruits, mehandi leaves powder and chopped fresh green chili were used for the preparation of methanolic extract.

The plant extracts were prepared according to prescribed method (Sirohi *et al.*, 2009) with some modifications. Plant extracts were prepared in aqueous methanol (water, methanol 50/50 ml).

A total of 25 g powder and chopped green chili in 500 ml conical flask were mixed with 250 ml 50% aqueous methanol (1:10 material to solvent). The flask was tightly sealed and kept in a shaker at 25°C and 120 rpm for 24 h.

After shaking, the contents of the flask were squeezed through four layers of muslin cloth and then filtered through Whatman No. 1 filter paper. The residue was re-extracted with 125 ml solvent under the same conditions and filtered through Whatman No. 1 filter paper. Extracts were combined and stored at 4°C for further use.

### Preparation of diet

To evaluate the effect of different methanolic plant extracts, two diets were prepared by selecting different roughages and concentrates in the ratio of 80 : 20 (HFD, high fiber diet) and 20 : 80 (LFD, low fiber diet). Details of physical compositions of diets are presented in Table 1.

**Table 1:** Ingredients of sorghum based diets used as substrate in *in vitro* incubation.

Roughage	
Particulars	g/kg on DM basis
Wheat straw	700
Sorghum fodder	300

Concentrate	
Particulars	g/kg on DM basis
Maize	330
Ground nut cake	210
Mustard cake	120
Wheat bran	200
Deoiled rice bran	110
Mineral mixture	20
Salt	10

### Treatments and experimental design

One milliliter of each extract was added to the diet sample in a glass syringe (100 ml) containing 200±10mg of milled sorghum based high and low fiber diets.

All treatments were arranged in factorial arrangement (2×4) in randomized block design with three replicates. Sets were also incubated devoid of substrate with and without plant extracts, which served as blanks for particular treatments with extract and control diet and the values were corrected accordingly for different parameters with these blanks.

### In vitro gas production

The incubation medium was prepared as described by Menke and Steingass (1988). Rumen liquor was collected from a fistulated male buffalo (*Bubalus bubalis*) maintained on a standard diet (60 parts roughage : 40 parts concentrate) before morning feeding into an insulated flask. The rumen liquor was filtered through four layers of muslin cloth and then the required amount of filtered rumen liquor was used as a source of inoculum.

One milliliter of each extract was added to the diet sample in glass syringe (100ml) containing 200±10 mg of milled (1mm) two type sorghum based diets (HFD and LFD). Syringes were incubated at 39±0.5°C for 24 h. Petroleum jelly was applied on the plungers of syringes for smooth movement and to stop any leakage. Syringes were closed using clamps. Syringes were incubated for 24 h in case of *in vitro* dry matter digestibility (IVDMD), total volatile fatty acids (TVFAs), individual volatile fatty acids (IVFAs) and methane, while a different set was incubated for 96 h (in sequential incubation for 0, 1, 2, 3, 6, 9, 12, 24, 36, 48, 60, 72 and 96 h) in case of gas kinetics study.

### Total gas production and methane estimation

After 24 h incubation, total gas production was calculated by subtracting gas produced in blank syringe (containing no substrate, but only the inoculum and buffer) from total gas produced in the syringe containing substrate, inoculum and buffer. Methane content in fermentation gas was determined by gas chromatography (GC) using Nucon-5765 gas chromatograph equipped with a flame ionization detector (FID) and a stainless steel column packed with Porapak-Q (length 6’;o.d.1/8” i.d. 2 mm; mesh range 80-100).

Temperatures were 40, 50 and 50°C, in injector oven, column oven and detector, respectively and the flow rates of carrier gas (nitrogen), hydrogen and air were 30, 30 and 300 ml/min, respectively. For methane estimation, each gas sample (250µl) was manually injected using Hamilton airtight syringe. Methane content in sample was calculated by external calibration, using a certified gases mixture with 50% CH_4_ and 50% CO_2_ (Spantech calibration gas, Surrey, England). The volume of methane produced was calculated as follows:

Methane production (ml) = Total gas produced (ml) × % methane in the sample.

### Partitioning factor and microbial biomass yield

The partitioning factor (PF) is calculated as the ratio of substrate (truly degraded dry matter) *in vitro* (mg) to the volume of gas (ml) produced by it.

The Microbial Biomass (MBM) yield was calculated by using the degradability of substrate and gas volume and stoichiometrical factor (Blummel *et al.*, 1997).

Microbial mass (mg) = Substrate truly degraded - (Gas volume × Stoichiometrical factor).

Where, the stoichiometrical factor used was 2.25.

### Total and Individual volatile fatty acid estimation

TVFA concentration (mM/100ml) in the supernatant was estimated as previously described (Barnet and Reid, 1957).

For IVFA at the end of incubation (24 h), 1 ml of the supernatant was treated with 25% meta-phosphoric acid (4 ml) and kept for 3-4 h at ambient temperature (Erwin *et al.*, 1961). Thereafter, percent IVFA was estimated by gas chromatography according to the described method by Sirohi *et al*. (2012) and calculations were performed for mM per 100 ml using TVFA concentration.

### Estimation of ammonia nitrogen

The supernatant in each syringe including that of the blank was used for NH_3_-N estimation. Supernatant (5 ml) was mixed with 1 N NaOH (2 ml) and steam passed using KEL PLUS - N analyzer (Pelican, India) and the NH_3_ evolved was collected in boric acid solution with a mixed indicator and titrated against N/100 H_2_SO_4_.

### Protozoa counting

The protozoa in fermentation fluid were counted on a haemocytometer as per the method described by Dehority (1984).

### In vitro true dry matter degradability

To estimate true dry matter (DM) degradability of feed, sample of each syringe containing residues after incubation was estimated as per the prescribed method (Van Soest *et al.*, 1991).

### Proximate analysis and cell wall constituents

The proximate analysis (organic matter, crude protein, ether extract and total ash) of substrate was carried out as per the methods of AOAC (1995). The Neutral detergent fiber of substrates were determined according to prescribed method of Van Soest *et al*. (1991) and other cell wall components such as acid detergent fiber (ADF) and hemicellulose (HC) as per the method of Robertson and Van Soest (1981).

### Gas production kinetics

The total gas production kinetics was carried out in methanolic plant extracts in a separate set containing three replicates as per procedure mentioned above for different intervals i.e. 0, 1, 2, 3, 6, 9, 12, 24, 36, 48, 60, 72 and 96 h. The potential gas production and rate of gas production was calculated by fitting the modified equation (Orskov and McDonald, 1979). All data of gas production were analyzed (analysis of variance) using factorial design in randomized block design.

Regression model = Orskov without lag.

Equation:

F= b*(1-exp (-c*x))

*a = is not taken in modified equation because it is assumed that the gas production in different samples or treatment combinations starts instantly and not having any lag phase.

### Statistical analysis

Experimental data of different parameters were analyzed in 2×4 factorial arrangements in randomized block design with three replicates for analysis of variance (Snedecor and Cochran 1968).

The effects of different diet and methanolic extracts (treatments) compared with controls were tested using the factorial arrangement in complete randomized block design in OPSTAT statistical software developed by Chaudhry Chran Singh, Haryana Agriculture University, Hissar, Haryana, India (Sheoran, 2010).

When the overall *F*-test was significant, differences between means and the control were declared significant at *P*≤0.05 using the Fisher’s-Least-Significant-Difference (Critical Difference) and Standard Error of Means (SEM) values were presented.

## Results

Physical and chemical composition of sorghum based high and low fiber-containing diets used as substrate for *in vitro* incubation was shown in Tables 1 and 2.

**Table 2:** Chemical composition of Sorghum based diets used as substrate in *in vitro* incubation.

Diets	Chemical constituents (% DM basis)						
	OM	CP	EE	NDF	ADF	HC	TA
HFD (80R:20C)	89.3	11.6	1.85	57.5	39.1	18.4	10.7
LFD (20R:80C)	90.2	19.6	3.52	27.9	19.2	8.7	9.8

*Values are mean of three samples

HFD=High Fiber Diet, LFD= Low Fiber Diet, OM= Organic Matter, CP= Crude Protein, EE= Ether Extract, NDF= Neutral Detergent Fiber, ADF= Acid Detergent Fiber, HC= Hemicelluloses, TA= Total Ash.

Results of different methanolic plant extract on methane production and other nutritional parameters are presented in Tables 3 and 4.

**Table 3:** Effect of addition of methanolic extracts on digestibility, methane production and other rumen parameters in sorghum based diets

Diet	Treatment	Parameters						
		pH	DDM (mg)	PF	MBM (mg)	CH_4_ (mM/g DM)	NH_3_-N (mg/100ml)	Protozoa (x10^4^/ml)
HFD	Control	6.91	117.00	3.97	50.63	2.35	3.27	1.00
	T_1_	6.89	115.67	2.92	26.42	2.93	4.12	1.00
	T_2_	7.06	102.33	4.45	50.58	1.97	4.57	0.83
	T_3_	7.04	114.33	4.72	59.58	2.19	5.04	0.67

LFD	Control	6.94	141.67	3.82	57.67	2.57	6.81	1.17
	T_1_	6.89	126.00	2.83	27.65	3.34	4.01	1.17
	T_2_	7.04	118.33	4.55	68.96	2.01	4.09	0.67
	T_3_	6.95	131.33	3.66	50.33	3.23	6.63	1.17

SEM	D	NS	2.08	NS	NS	0.08	0.06	NS
	T	0.01	2.40	0.14	3.46	0.09	0.07	NS
	D x T	NS	4.16	NS	NS	NS	0.13	NS

HFD=High Fiber Diet, LFD= Low Fiber Diet, T_1_= Mehandi, T_2_= Jaiphal, T_3_= Green Chili, DDM= Digestible Dry Matter, PF= Partition Factor, MBM= Microbial Biomass, CH_4_= Methane, NH_3_-N= Ammonia Nitrogen, SEM= Standard Error of Means.

**Table 4:** Effect of methanolic extracts of mehandi, jaiphal and green chili on short chain fatty acids on sorghum based high and low fiber diet.

Diet	Treatment	Parameters				
		TVFA (mM/100ml)	Acetate (mM/100ml)	Propionate (mM/100ml)	Butyrate (mM/100ml)	A/P ratio
HFD	Control	5.17	3.92	0.94	0.30	4.18
	T_1_	4.60	3.33	0.90	0.37	3.73
	T_2_	4.87	2.90	0.76	0.31	3.81
	T_3_	4.72	3.50	0.89	0.33	3.96

LFD	Control	5.60	4.09	1.13	0.38	3.63
	T_1_	4.42	3.22	0.88	0.32	3.68
	T_2_	4.93	3.06	0.96	0.41	3.19
	T_3_	5.40	3.91	1.07	0.42	3.64

SEM	D	NS	NS	0.04	0.01	0.07
	T	0.22	0.16	0.04	NS	NS
	D*T	NS	NS	NS	NS	NS

T_1_= Mehandi, T_2_= Jaiphal, T_3_= Green Chili, TVFA= Total Volatile Fatty Acids, A/P= Acetate to Propionate Ratio, SEM= Standard Error of Means.

Effect of methanolic extract on pH was not significant for all three diets except mehandi extract treatment. Maximum 0.15 unit variation was found in different treatment combinations. Maximum pH was found in T_2_ (7.06) in HFD and minimum was in T_1_ (6.89) in HFD and LFD, respectively.

In present study, DDM (mg) was significantly affected by plant extracts. The DDM was decreased significantly (16.47%) due to addition of T_2_, in case of LFD, while T_2_ also caused a 12.53% reduction in DDM in case of HFD.

However, mehandi (T_1_) and chili (T_3_) extract shown very little effect on DDM in comparison to jaiphal (T_2_). A reduction in methane production (mM/gDM incubated) was induced by T_2_ in all diet in comparison with control diet without addition of extract, while T_1_ and T_3_ did not induce any significant effect on methane reduction.

Results indicated that the maximum methane reduction was observed in jaiphal (T_2_), i.e. T_2_ reduced methane production 21.78 and 16.17% on the addition in LFD and HFD, respectively. A non-significant (P≤0.05) effect of different plant extracts was observed on protozoal number. However, apparently among the different treatments, T_2_ showed the maximum reduction in protozoal number, i.e. 42.73% LFD.

Acetate to propionate ratio was not affected in different treatment combinations with addition of plant extracts, but diets had shown significant effect on A : P ratio. The maximum reduction A : P ratio was found to be around 12.12% on the addition of T_2_ to LFD.

The reduced protozoal number is sometimes associated with increase in propionate and decrease in A : P ratio. Again, T_2_ had shown the highest reduction of A : P ratio. In the current study, T_1_ significantly reduced the PF and MBM (mg) in all diets, while PF and MBM (mg) were not affected on the supplementation of T_2_ and T_3_.

However, in case of LFD with supplementation of T_2_, PF and MBM were increased up to 19%. Results showed that TVFA concentration decreased with inclusion of methanolic extracts in all the three diets and T_2_ shown the highest (25.18%) reduction in case of LFD, while in case of HFD, T_1_ shown the highest 11.02% reduction.

Overall production of IVFA was decreased due to the addition of methanolic extracts, and this is because of the digestibility of dry matter, which was also decreased in most of the cases. The acetate concentration (mM) was decreased with plant extracts in all the three diets. It was decreased (26.02 and 25.18%) maximum with T_2_ in both diets. Propionate concentration was also decreased with T_2_ (19.14%) in HFD and (15.04%) in LFD, respectively. Butyrate concentration remained similar and non-significant in the present study.

Results of plant extracts addition on gas kinetics are presented in Table 5. It was observed from the results that potential gas production (b) was increased due to the supplementation of plant extracts, and the increase was highest (102.52%) in T_3_ treatment in HFD diet. The gas production rate constant (c) decreased after supplementation of plant extracts, and the reduction was highest (77.27%) on the addition of T_2_ in case of HFD.

**Table 5:** Effect of methanolic extracts of mehandi, jaiphal and green chili on gas kinetics on sorghum based high and low fiber diet.

Diet	Treatments	b	c	R^2^
HFD	Control	49.99	0.044	0.984
	T_1_	53.48	0.035	0.998
	T_2_	86.81	0.010	0.993
	T_3_	101.24	0.015	0.994

LFD	Control	43.32	0.075	0.996
	T_1_	59.56	0.067	0.996
	T_2_	63.85	0.021	0.995
	T_3_	70.48	0.034	0.991

b = Potential Gas Production (ml), c = Gas Production Rate Constant (ml/h), R^2^ = Regression Coefficient.

## Discussion

Methane is produced in rumen by enteric fermentation is nutritionally wasteful process which represent 2 to 15% feed energy loss (Moss, 1993) and ruminants involve 15 to 20% of the global production of methane (Crutzen *et al.*, 1986). Therefore, reduction of methane production exerts significant economic and environmental benefits.

In present study, Methane production either not affected due to T_1_ or T_3_ supplementation or decrease significantly by T_2_ (jaiphal) supplementation and protozoa number also decreased by T_2_. This tendency to reduce methane emission by plant extract might be due to inhibition of protozoal population directly as reported in other studies by plant extracts and PSM (Guo *et al.*, 2008).

As some of the PSM like saponins are act as a defaunating agent and break the association of ciliate protozoa and methanogen which indirectly helps in decreasing the methane production (Patra and Saxena, 2010).

In current study, decrease in TVFA, acetate concentration (mM/100 ml), slight change in propionate concentration and decrease in A : P ratio was observed in supplementation with methanolic extract, which is in accordance with previous study done by Hess *et al.*, 2003, Hristov *et al.*, 2003 and Patra *et al.*, 2006.

A large number of plant extracts and spices were evaluated earlier for their anti-methanogenic activities. Nature, activity and concentration of the active components present reflects, what type of effect a particular plant species will have on methanogenesis (Gonzalez *et al.*, 2008).

The exact mechanism of action of active compound present in different plants or their products is unknown in the large plant kingdom.

However, the effects of certain PSM have been reviewed recently in detail by Patra and Saxena (2010). The present study enriched our knowledge about the anti-methanogenic activity of these plant extracts.

## Conclusion

It was concluded from the present study that jaiphal (*Myristica fragrans*) has anti-methanogenic potential without affecting the major nutritional parameters. However, further studies are required to further explore the active components present in jaiphal, different dosages and mechanism of action on methanogenesis.
